# Autologous bone grafting combined with spheroid‐based matrix‐induced autologous chondrocyte implantation for osteochondral defects of the knee: Good clinical outcomes alongside abnormal postoperative gait patterns

**DOI:** 10.1002/ksa.12605

**Published:** 2025-02-04

**Authors:** Stephan Oehme, Danko Dan Milinkovic, Azzurra Paolucci, Sophie Krafzick, Stephen Fahy, Philipp Damm, Tobias Winkler, Tobias Jung, Benjamin Bartek

**Affiliations:** ^1^ Charité ‐ Center for Musculoskeletal Surgery, Universitätsmedizin Berlin, Corporate Member of Freie Universität Berlin Humboldt‐Universität zu Berlin Berlin Germany; ^2^ IRCCS Istituto Ortopedico Rizzoli di Bologna Università Degli Studi Alma Mater Studiorum di Bologna Bologna Italy; ^3^ Berlin Institute of Health at Charité, Julius Wolff Institute Universitätsmedizin Berlin Berlin Germany; ^4^ Berlin Institute of Health Center for Regenerative Therapies, Berlin Institute of Health at Charité Universitätsmedizin Berlin Berlin Germany

**Keywords:** ABCI, ACI, autologous bone grafting in combination with autologous chondrocyte implantation, autologous chondrocyte implantation, cartilage, MACI

## Abstract

**Purpose:**

This study aimed to evaluate the clinical and functional outcomes of autologous bone grafting with spheroid‐based matrix‐induced autologous chondrocyte implantation (MABCI) for osteochondral defects of the knee by analysing pre‐ and postoperative patient‐reported outcome measures (PROMs). Postoperative gait analysis was conducted and compared with a matched healthy control group to investigate biomechanical deviations.

**Methods:**

A total of 35 patients (m: 21, f: 14; mean defect size: 4.2 ± 2.4 cm², localisation: femoral condyle: 31, patellofemoral: 5) were analysed. The mean follow‐up was 42.6 ± 22.8 months. International Knee Documentation Committee (IKDC), Knee Injury and Osteoarthritis Outcome Score (KOOS), PROMIS 29 profile, and a questionnaire on patient perception of treatment success were assessed to evaluate PROMs. 3D‐instrumented gait analysis (GRAIL, Motek) was used to assess lower extremity kinematics, kinetics and vertical ground reaction forces, compared to sex‐, age‐ and body mass index‐matched healthy controls.

**Results:**

All clinical scores showed significant improvement compared to the preoperative condition (IKDC: 73.1 ± 10.1 vs. 56.6 ± 17.2, *p* < 0.01; KOOS subcategories: pain 82.0 [±12.7] vs. 70.7 [±16.7] [*p* < 0.01], symptoms 79.1 [±20.3] vs. 68.9 [±13.9] [*p* < 0.01], activities of daily living 90.1 [±11.2] vs. 80.5 [±15.6] [*p* < 0.01], sport and recreational function: 65.3 [±19.3] vs. 51.3 [±26.29] [*p* < 0.01], quality of life 52.2 [±18.6] vs. 42.6 [±18.6] [*p* < 0.01]; numeric pain rating scale: 2.7 ± 2.0 vs. 5.0 ± 2.5, *p* < 0.01). The analysed patients reported a high satisfaction rate (94.3%). Self‐selected walking speed was significantly lower than in healthy controls (1.17 ± 0.17 m/s vs. 0.98 ± 0.18 m/s, *p* < 0.01). Peak knee flexion angle (PKA) during loading response was significantly smaller (9.6° ± 7.0 vs. 17.7° ± 4.6, *p* < 0.01), and knee extension moment was significantly reduced (0.1 Nm/kg ± 0.2 vs. 0.4 Nm/kg ± 0.2, *p* < 0.01).

**Conclusion:**

MABCI is an effective treatment for osteochondral knee defects, showing significant improvements in all evaluated PROMs. Postoperative gait analysis revealed abnormal gait patterns, including reduced PKA and lower knee extension moment, suggesting a need for further rehabilitation to optimise functional recovery.

**Level of Evidence:**

Level III.

**Abbreviations**
ABCI: autologous bone grafting in combination with autologous chondrocyte implantation
GACI: gel‐based autologous chondrocyte implantation
GRAIL: Gait Real‐time Analysis Interactive Laboratory system
HBM: human body model
HS: heel strike
IKDC: International Knee Documentation Committee
KOOS: Knee Injury and Osteoarthritis Outcome Score
MABCI: autologous bone grafting in combination with matrix‐induced autologous chondrocyte implantation
OAT: osteochondral autograft transplantation
PKA: peak knee flexion angle
PROMs: patient‐reported outcome measures
TKA: total knee arthroplasty
VGRF: vertical ground reaction force
WA: weight acceptance

## INTRODUCTION

Untreated osteochondral defects can lead to joint degeneration and osteoarthritis [13, 37, 40]. Over the years, various surgical techniques have been developed and refined to treat cartilage defects [20]. Treatment options for chondral lesions include bone marrow‐stimulating procedures like microfracture or autologous matrix‐induced chondrogenesis, as well as autologous chondrocyte implantation (ACI) and matrix‐associated chondrocyte implantation (MACI). Advanced MACI forms, such as hydrogel‐ and spheroid‐based approaches, offer injectability, eliminate the need for additional fixation and show remarkable histological results [7, 14, 18]. These techniques are limited to lesions without subchondral pathologies [24, 29, 36]. Osteochondral defects require combined cartilage and subchondral bone treatment, making the ideal procedure selection particularly challenging. Osteochondral autograft transplantation is effective for small defects but controversial for larger defects due to donor site morbidity [17]. To address these limitations, autologous bone grafting combined with autologous chondrocyte implantation (ABCI), first described by Peterson et al. as the ‘sandwich technique’, has been developed [33]. A recent systematic review showed significant improvements in Patient‐Reported Outcome Measures (PROMs) following ABCI for the treatment of osteochondral defects, with reduced pain, better knee function and increased activity levels [31]. The assessment of clinical outcomes of ABCI remains challenging due to the reliance on older studies with small cohorts and considerable variability in surgical techniques. Moreover, no studies have utilised gait analyses to evaluate functional recovery in these patients. To address these gaps, our study assessed for changes in both pre‐and postoperative PROMS as well as a postoperative gait analysis in a patient cohort treated with a standardised ABCI procedure, utilising an advanced third‐generation spheroid‐based matrix‐induced autologous chondrocyte implantation combined with autologous bone grafting (MABCI). The main objective of the present study was to evaluate the clinical outcomes of MABCI by analysing pre‐ and postoperative PROMs. A secondary outcome of interest was the comparison of postoperative gait with that of a matched healthy control group. It was hypothesised that MABCI leads to significant improvements in postoperative PROMs compared to the preoperative status. Additionally, it was hypothesised that patients after MABCI would show no differences in gait analysis regarding spatiotemporal parameters, vertical ground reaction force (VGRF), knee kinematics and knee kinetics compared to a matched healthy control cohort.

## MATERIALS AND METHODS

### Study design and patient cohort

A retrospective cohort analysis of prospectively collected data was conducted. Between September 2014 and August 2020, 68 patients underwent MABCI as a treatment for osteochondral defects of the knee at a single university centre. (Center for Musculoskeletal Surgery, Charité—Universitaetsmedizin Berlin, Berlin, Germany). Out of the 68 cases, four patients did not meet the inclusion criteria and were excluded from the final analysis (Table 1). Two patients presented with meniscal injuries involving more than one‐third of the meniscus at the time point of surgery. One patient had a preoperatively diagnosed neoplastic condition and another required a revision surgery that involved conversion to the implantation of a customised metal implant. All patients undergoing MABCI received a preoperative long‐leg standing AP x‐ray. Patients with tibiofemoral malalignment exceeding three degrees from the neutral mechanical axis toward the affected compartment were treated with concomitant osteotomy in the second surgery [1].

**Table 1:** Inclusion and exclusion criteria patient cohort.

Inclusion	Age ≥18 years
	At the time of surgery, symptomatic osteochondral defect International Cartilage Regeneration & Joint Preservation Society grade 4 of the knee, confirmed by MRI imaging and arthroscopic findings
	Surgical treatment with a combination of autologous bone grafting and spheroid‐based matrix‐induced autologous chondrocyte implantation
	Follow‐up at least 1 year postoperatively
	Written informed consent
Exclusion	Meniscal injury of the affected knee involving more than 1/3 of the meniscus substance
	Ligamentous insufficiency of the affected or contralateral knee
	Condition after fracture of the affected or contralateral limb
	Conversion to total or partial knee replacement
	Patients with neoplastic diseases
	Pregnancy

**Figure 1:**
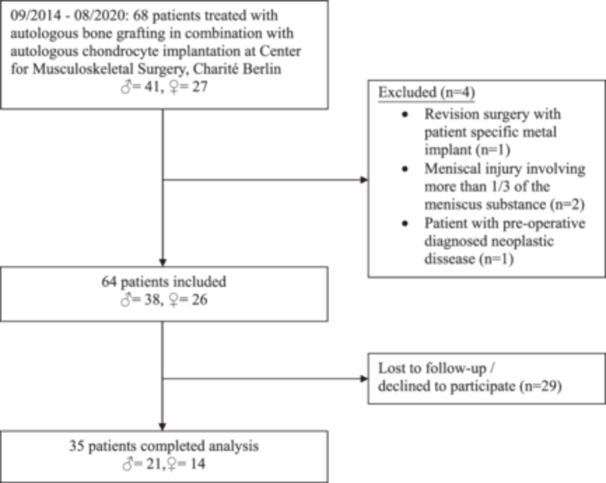
Consort diagram of patient acquisition.

Patients were invited for a comprehensive clinical and biomechanical assessment. Follow‐up was not feasible in 29 cases due to changes in address, unavailable contact information or the patient's decision to decline participation, resulting in a loss to follow‐up. Consequently, 35 patients were included in the final analysis. A detailed flowchart is provided in Figure 1.

A healthy body mass index‐, sex‐ and age‐matched control group was recruited for comparison of the gait analysis. All subjects in the control group had no musculoskeletal complaints or history of musculoskeletal surgery. The inclusion and exclusion criteria of the control group are listed in Table 2. The matched control group served as a healthy control group to detect postoperative gait deviations in comparison to the analysed patient cohort.

**Table 2:** Inclusion and exclusion criteria control group.

Inclusion	Age ≥18 years
	No history of musculoskeletal surgery
	No musculoskeletal complaints for the last 6 months
	No neurological diagnoses, comorbidities or other physical limitations that affect the gait pattern
	Not currently undergoing orthopaedic or rheumatology treatment
	No known degenerative musculoskeletal conditions
	No current nonmusculoskeletal symptoms affecting gait (such as fatigue, cough, fever)
	Written consent
Exclusion	Known, suspected or history of rheumatologic disease
	Known, suspected or history of neurologic disease
	Known, suspected or history of neoplastic diseases
	Pregnancy

### Surgical technique

**Figure 2:**
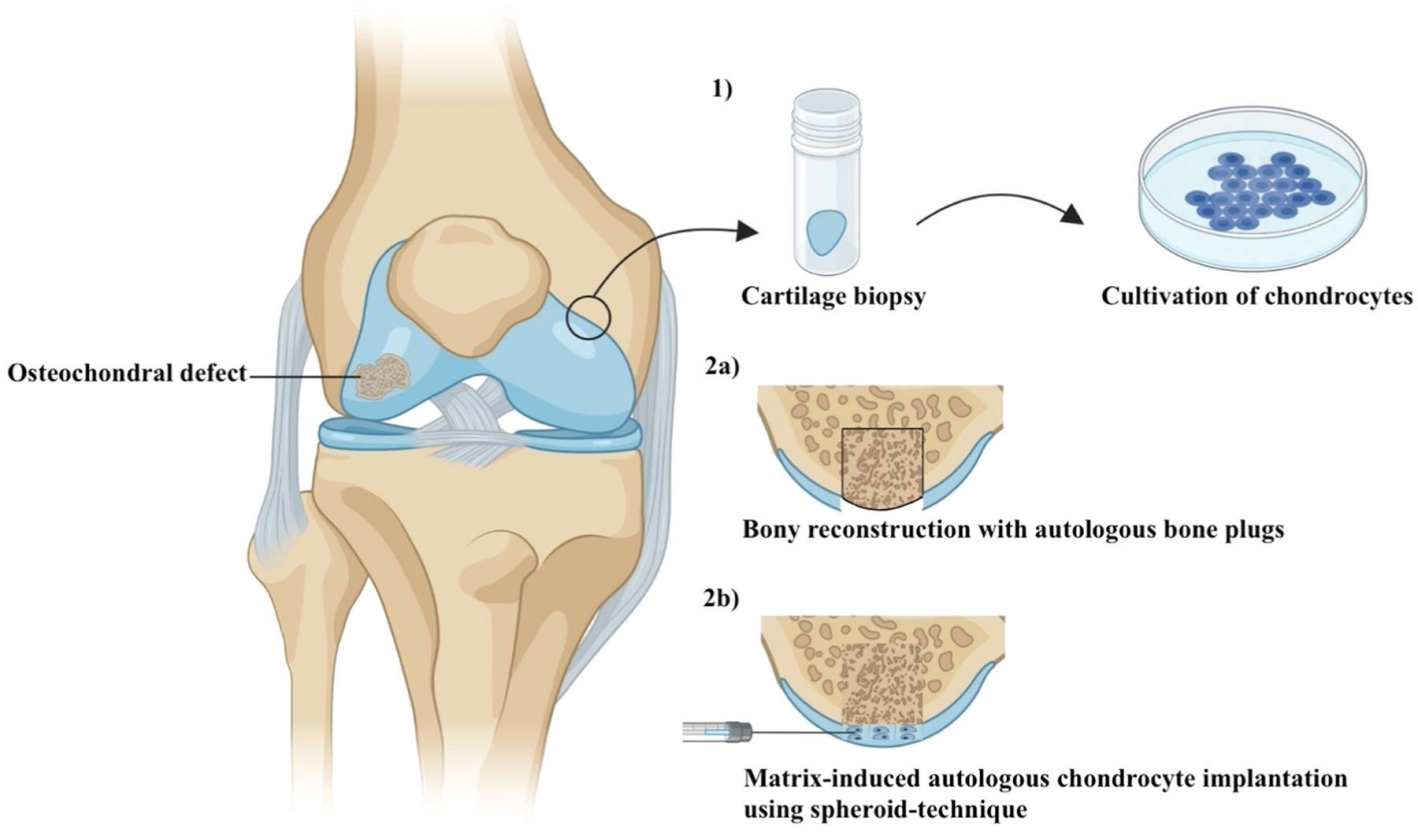
Procedure of autologous bone grafting in combination with spheroid‐based matrix‐induced autologous chondrocyte implantation.

All patients received MABCI as a treatment for osteochondral defects of the knee in a two‐step procedure (Figure 2).

**Figure 3:**
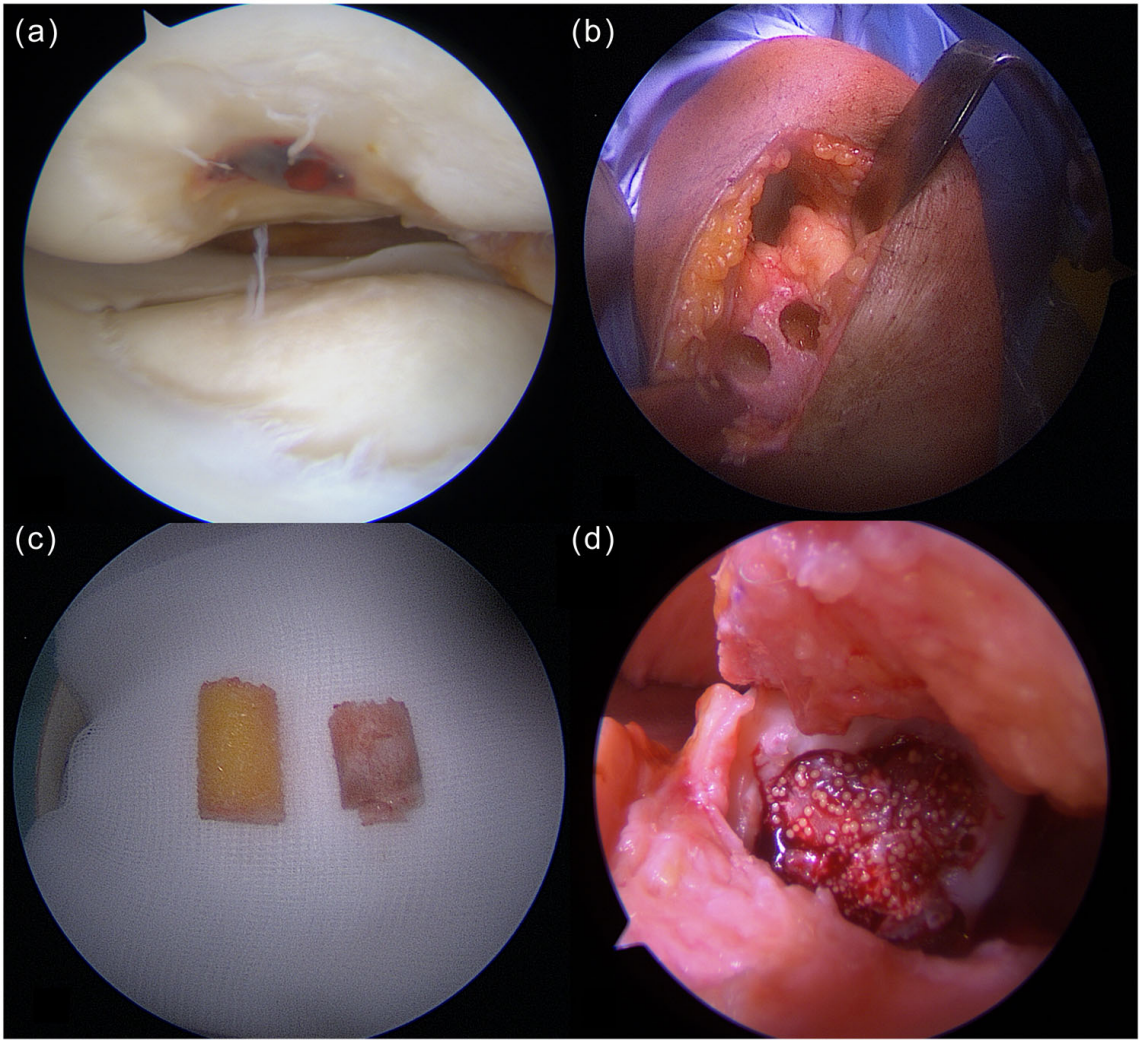
Intraoperative images of the surgical procedure. (a) Arthroscopic view of the osteochondral defect of the medial femoral condyle. (b) Harvest sites for the healthy corticocancellous bone plugs from the metaphysial region of the proximal tibia. (c) Harvested healthy corticocancellous bone plugs (left) and nonviable bone from the osteochondral defect (right). (d) Intraoperative Image at the end of the surgery with a reconstructed bony surface and implanted spheroid‐based matrix‐induced autologous chondrocytes.

The initial surgery consisted of a diagnostic arthroscopy to determine the extent and location of the osteochondral defect, as well as an arthroscopic assessment for concomitant injuries. Following the arthroscopic assessment of lesion size, location and depth, two to three five millimeter diameter osteochondral cylinders were harvested from the nonweight‐bearing intercondylar region of the femur. The samples were sent to the laboratory (CO.DON AG) for cultivation [5]. The resulting spheroids comprised spherical aggregates of the autologous chondrocytes and their extracellular matrix. Autologous bone grafting in combination with spheroid‐based matrix‐induced autologous chondrocyte implantation was performed 35–55 days after the initial surgery. The subsequent operation was performed through a mini‐arthrotomy approach. The defect was visualised and the damaged articular cartilage was debrided, with loose or diseased tissue surrounding the lesion being removed to facilitate optimal graft integration. Sclerotic and necrotic bony areas at the base of the chondral lesion were removed using an osteochondral autograft transfer system (Arthrex). Subsequently, healthy corticocancellous bone plugs were harvested from the metaphysial region of the proximal tibia or distal femur using the same osteochondral autograft transfer system. Depending on the lesion size, one to five corticocancellous bone plugs with a diameter of 10 millimeters each were harvested. The donor site was filled with allogenic cancellous bone. The harvested autologous corticocancellous bone plugs were then inserted into the defect area to create a healthy, stable and vascularised bed. The spheroids were gradually applied onto the corticocancellous bone plugs and evenly distributed. After complete application, the knee joint was immobilised to ensure adequate adhesion of the spheroids (Figure 3).

All patients underwent a structured and standardised rehabilitation protocol with regular outpatient physiotherapy to promote graft healing and functional recovery. For patients with an osteochondral defect of the femoral condyle, active and passive knee flexion was limited to 60**°** during the first 2 weeks, followed by a limitation of active range of motion to 90**°** flexion until the end of the 6th week. The range of motion for passive knee flexion was unrestricted for these patients starting from the 2nd week. For patients with a patellofemoral defect, active knee flexion was limited to 30**°** during the first 6 weeks postoperatively, followed by an increase to 90**°** in Weeks 7 and 8 postoperatively, with the limitation being removed after the 8th week postoperatively. Passive knee flexion was limited to 30**°** for these patients in the first 2 weeks and then gradually increased by 30**°** every 2 weeks. Patients with restored defects of the femoral condyle underwent 6 weeks of partial weight‐bearing, while those with patellofemoral region defects had 4 weeks of partial weight‐bearing.

### PROMs

The following PROMs have been used to assess the preoperative and postoperative status: Knee Documentation Committee Subjective Knee Function Score (IKDC) [21], the Knee Injury and Osteoarthritis Outcome Score (KOOS) [35] and the PROMIS‐29 Profile v2.1 [8]. Additionally, a short satisfaction survey was conducted with the following questions: How would you rate the result of your operation? (a: good; b: satisfatory; c: poor); If you had to make the decision again, would you opt for this procedure again? (a: yes; b: unsure; c: no).

### Range of motion and thigh and calf circumference

The circumferences of the thigh and calf, as well as the active range of motion of the operated legs, were measured and compared with those of the contralateral legs. Thigh circumferences were measured at 20 and 10 cm above the upper patellar pole, while calf circumferences were recorded at the point of maximum calf girth. Active range of motion for knee flexion and extension was evaluated in the sagittal plane using a goniometer, with patients positioned in a prone posture.

### Gait analysis

**Figure 4:**
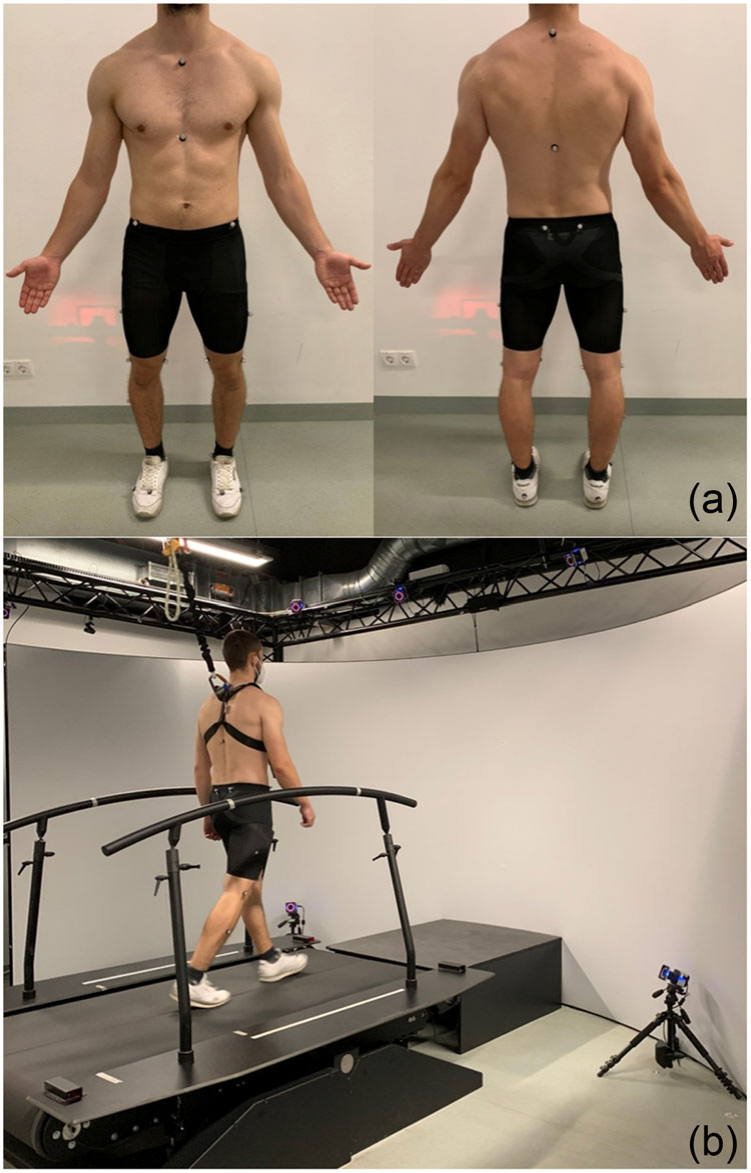
(a) Marker set of motion gait analysis to determine three‐dimensional kinematics and kinetics. (b) Motion gait analysis performed at a self‐selected speed on a treadmill with two force plates without a virtual reality environment (GRAIL, Motek Medical B.V., Amsterdam, Netherlands).

Gait analyses were performed on all subjects to determine three‐dimensional kinematics and kinetics at a self‐selected speed. A reflective marker set consisting of 26 markers was attached to the subjects' bodies according to the human body model (HBM) as shown in Figure 4a [12].

The reflective markers were captured by a 3D motion measurement system with infrared cameras (Vicon Metrics Ltd.). The Vicon system has excellent precision and high accuracy for low‐ to high‐speed experiments with a system error of <2 mm [27]. The Gait Real‐time Analysis Interactive Laboratory system (GRAIL) was used to carry out gait analysis. GRAIL includes a fully instrumented treadmill equipped with two force plates and a self‐paced option (Gait Real‐time Analysis Interactive Lab, GRAIL, Motek Medical B.V.; Figure 4b). The GRAIL system uses the HBM, a validated model developed for real‐time biomechanical analysis kinematics and kinetics analysis [12]. Differing from conventional models that calculate each segment independently using at least three markers per segment, the inverse kinematics in HBM are calculated simultaneously. Under real‐time speed settings, the kinematic errors resulting from premature termination of the iteration process are below 0.01° [41, 42]. The generalised forces are calculated through an inverse dynamic analysis, which has the low‐pass filtered kinematics and the ground reaction forces as input [19]. The GRAIL system has excellent test–retest reliability for spatiotemporal parameters and kinematic analysis and a high to excellent test–retest repeatability for kinetic analysis [2].

Before each measurement, standardised static and dynamic calibration recordings were made. During static calibration, HBM determines the segment reference frames based on the marker positions. For each real‐time frame, these segments are fitted onto the marker data by minimising the error between the marker data and the segment orientations. A functional calibration was performed to determine the joint centres. This was followed by a six minute familiarisation phase of walking on the treadmill. After familiarisation, three recordings of 30 s each at self‐selected speed were made. To avoid injuries resulting from accidental stumbling, subjects wore a safety harness. The study was approved by the local ethics committee (Ethikkommission der Charité—Universitätsmedizin Berlin; Nr: EA4/220/20) and was performed in accordance with the ethical guidelines of the Declaration of Helsinki. All subjects provided written informed consent before participation and were properly informed about the measurement procedures.

### Data analysis

The biomechanical parameters were analysed using MATLAB (version R2022b, The MathWorks Inc.). The respective peak values for kinematics, kinetics and VGRF were determined and then averaged. Mean values of step length, step width, stance time and swing time were calculated for the operated and contralateral legs of the patients. In the control group, results from both legs were averaged. The resulting data were summarised in Microsoft Excel (version 16.78, Microsoft Corporation). Graphs of the biomechanical parameters were averaged and displayed using MATLAB (version R2022b, The MathWorks Inc.). Questionnaire results were transferred from Heartbeat (version 7.41.1, HRTBT Medical Solutions GmbH) to Microsoft Excel (version 16.78, Microsoft Corporation) and graphically illustrated using RStudio software (version 2023.03.1 + 446, Posit Software PBC). Statistical analysis was performed using IBM SPSS Statistics (Version 28.0, IBM). A priori power analysis was conducted for gait kinematic measurements, based on data from a previous study that examined knee kinematics during gait in patients following matrix‐induced autologous chondrocyte implantation [10]. Assuming a difference of 4° between the injured knees and the knees of the control cohort, a minimum sample size of 25 subjects was required to achieve a statistical significance of 0.05 with 80% power.

Mean values and standard deviations were calculated for descriptive analysis. The Shapiro–Wilk test was used to assess data for normal distribution. Differences in mean values were examined using the *t* test. The paired *t* test was applied to compare the operated and contralateral legs, while the unpaired *t* test was used to compare the operated or contralateral leg with the control group. For data not normally distributed, the Wilcoxon signed‐rank test was used for related samples and the Mann–Whitney U test for unrelated samples. The significance level was set at *p* < 0.05 for all tests.

## RESULTS

### Patient data

A total of 35 patients were available for follow‐up assessment. Patient details are shown in Table 3. The mean follow‐up was 42.6 ± 22.8 months and the mean defect size was 4.2 ± 2.4 cm^2^. A total of 36 osteochondral defects of the knee were treated, including 25 located in the medial femoral condyle, six in the lateral femoral condyle, two in the trochlea and three retropatellar. Autologous bone grafting was performed in 29 patients using autologous corticocancellous bone plugs from the tibia and in six patients using corticocancellous autologous bone plugs from the distal femur. The tibia was utilised in cases where the quantity of bone from the femur was anticipated to be insufficient or the incision location favoured the tibial harvesting.

**Table 3:** Patient details.

Patient (sex)	Age at surgery, years	Age at follow‐up, years	BMI	Defect location	Lesion size, cm^2^	Follow‐up, years	Concomitant surgery	Previous surgeries
1 (F)	26	27	24.9	Trochlea	2.6	1.2	TTO	‐
2 (F)	19	24	20.4	MFC	3	4.7	LBR	allogenic ABG
3 (F)	29	31	30	MFC	3.6	1.3	HTO	‐
4 (M)	42	45	27.2	MFC	6	3.3	HTO	‐
5 (M)	19	24	19.2	MFC	5	5.8	LBR	‐
6 (M)	24	25	26,5	LFC	3.5	1.6	‐	‐
7 (F)	50	55	21.7	MFC & LFC	3.5	4.8	‐	‐
8 (M)	35	36	20.5	MFC	1.8	1	LBR	‐
9 (M)	20	21	31	MFC	1.8	1.1	‐	‐
10 (M)	37	43	38	MFC	2	5.7	HTO	‐
11 (F)	18	19	25.8	MFC	3	1.1	‐	OAT
12 (M)	39	40	21.8	LFC	3.5	1	‐	‐
13 (F)	18	24	25.1	MFC	3.2	5.1	‐	‐
14 (F)	20	26	22.5	LFC	4	5.2	‐	‐
15 (M)	38	44	25.4	MFC	5.4	5.8	‐	‐
16 (F)	25	27	22	MFC	6	2.1	‐	‐
17 (F)	30	37	20	MFC	2	6.9	‐	OAT
18 (M)	18	21	27.1	RP	2	2.1	‐	MFX
19 (M)	28	30	29.9	MFC	3	2.1	LBR	MFX
20 (M)	17	19	24.1	Trochlea	5	1.2	LBR	ACT, LBR
21 (M)	22	27	22.8	MFC	3.8	5	‐	‐
22 (M)	40	45	26.5	MFC	4	5.8	‐	‐
23 (M)	15	18	20.8	RP	5	2.2	‐	‐
24 (F)	38	41	25.8	MFC	4.5	2.6	‐	LBR
25 (M)	27	31	21.9	MFC	4.5	4	LBR	MFX
26 (F)	19	21	23.8	MFC	6	2.3	‐	ABG
27 (F)	32	38	22.6	MFC	12.3	6.2	‐	‐
28 (F)	33	40	38.2	LFC	1.5	7.1	‐	OAT
29 (F)	16	19	24	MFC	7	3.3	LBR	‐
30 (M)	21	25	18.7	MFC	12	4.3	‐	MFX
31 (M)	16	20	22.3	MFC	5	4.5	‐	‐
32 (M)	28	33	30.6	RP	2.5	4.3	LBR	‐
33 (M)	43	48	35	MFC	3	4.6	‐	‐
34 (M)	39	42	25.3	MFC	1.5	3	ACI PF	‐
35 (M)	15	18	25.3	LFC	3.5	2.3	LBR	‐

Abbreviations: ABG, autologous bone graft; ACI, autologous chondrocyte implantation; BMI, body mass index; F, female; HTO, high‐tibial osteotomy; LBR, loose body removal; LFC, lateral femoral condyle; M, male; MFC, medial femoral condyle; MFX, microfracture; OAT, osteochondral autograft transplantation; PF, patellofemoral; RP, retropatellar; TTO, tibial tubercle osteotomy.

### Control group

For the comparison of the gait analysis, 35 healthy BMI‐ and age‐matched subjects were recruited (Table 4).

**Table 4:** Characteristics of patient cohort and control group.

Parameter	Patients (*n* = 35)	Control Group (*n* = 35)	*p* Value
Age at follow‐up, mean ± SD (range), years	31 ± 10.2 (18–55)	33.4 ± 9.6 (19–57)	0.303
Sex, male/female, *n*	21/14	21/14	1.0
Body mass index, mean ± SD (range), kg/m^2^	25.3 ± 4.8 (18.7–38.2)	24.1 ± 3 (18–32)	0.220

### Patient‐reported outcomes

**Figure 5:**
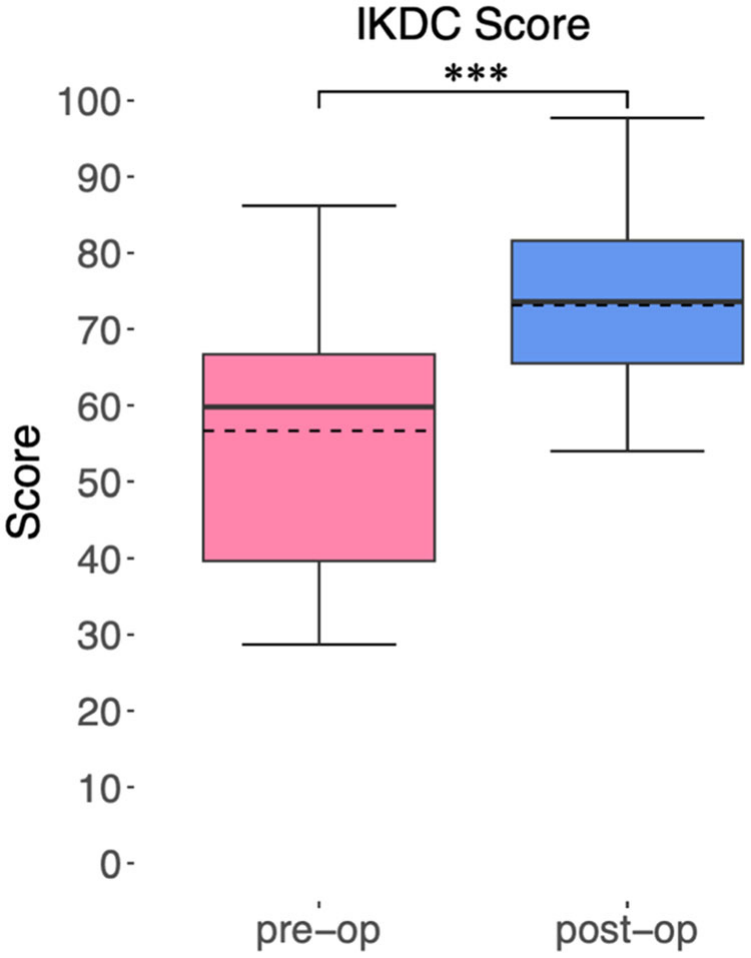
Subjective assessment of knee joint health using the subjective International Knee Documentation Committee (IKDC) score preoperatively and postoperatively (***p < 0.001).

The IKDC score improved significantly postoperatively from an average score of 56.6 (±17.2)–73.1 (±10.1), (*p *< 0.01) (Figure 5).

**Figure 6:**
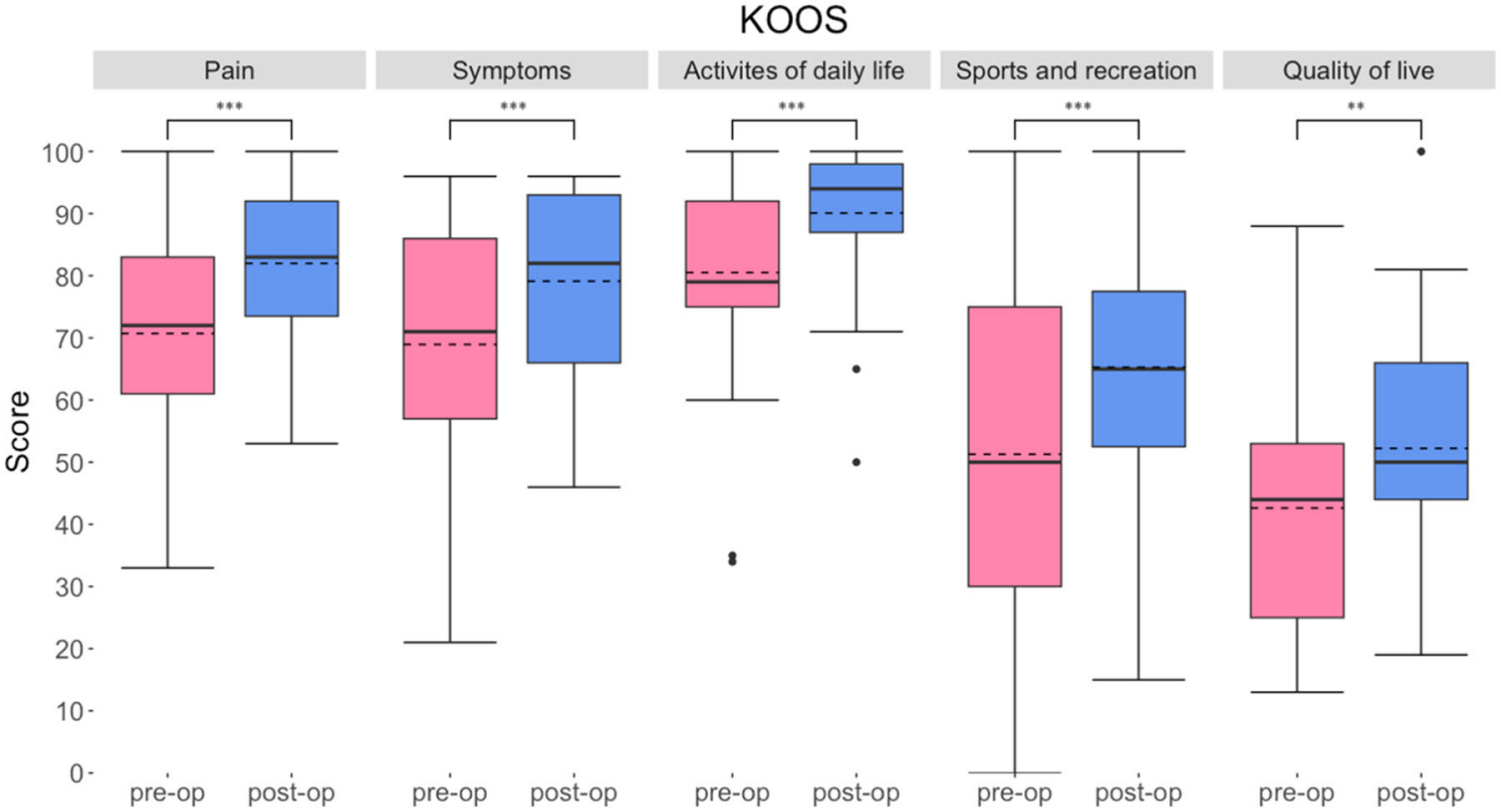
Subjective assessment of knee joint health using the Knee Injury and Osteoarthritis Outcome Score (KOOS) preoperatively and postoperatively (***p < 0.001).

All KOOS subcategories showed a significant improvement comparing the preoperative score with the postoperative score: Pain 70.7 (±16.7) versus 82.0 (±12.7) (*p *< 0.001), symptoms 68.9 (±20.3) versus 79.1 (±13.9) (*p *< 0.001), activities of daily living (ADL) 80.5 (±15.6) versus 90.1 (±1.2) (*p*< 0.001), sport and recreational function: 51.3 (±26.29) versus 65.3 (±19.3) (*p *< 0.001), quality of life 42.6 (±18.6)–52.2 (±18.6) (*p* = 0.002) (Figure 6).

**Figure 7:**
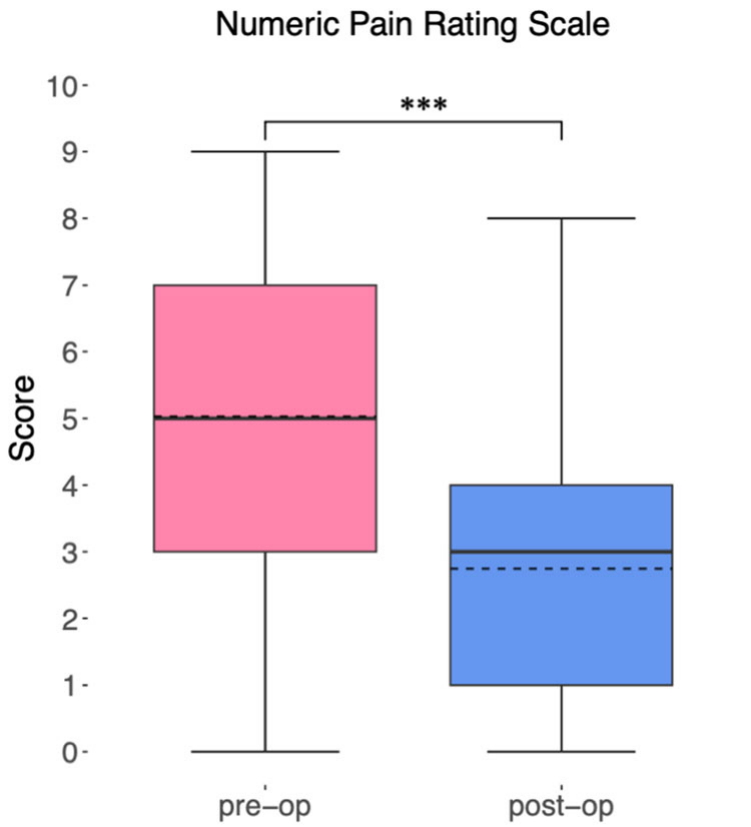
Preoperative and postoperative assessment of the numeric rating scale for pain intensity included in the PROMIS‐29 Profile v2.1 (***p < 0.001).

The evaluation of the PROMIS‐29 Profile v2.1 revealed notable findings across various domains. The pain impairment category demonstrated a significant decrease from 55.4 (±6.4) preoperatively to 49.9 (±6.6) postoperatively (*p *< 0.01). Furthermore, there was a significant improvement in pain intensity, with the numeric rating scale score decreasing from 5.0 (±2.5) preoperatively to 2.7 (±2) postoperatively (*p* < 0.01) (Figure 7). The physical functioning category exhibited a substantial improvement, with the mean score increasing from 45.2 (±6.7) preoperatively to 50.2 (±5.9) postoperatively (*p* < 0.01). Notably, there was a significant enhancement in participation in social roles and activities, as indicated by the increase from a preoperative mean score of 50.4 (±8.5) to 54.5 (±6.5) postoperatively (*p* = 0.01).

**Figure 8:**
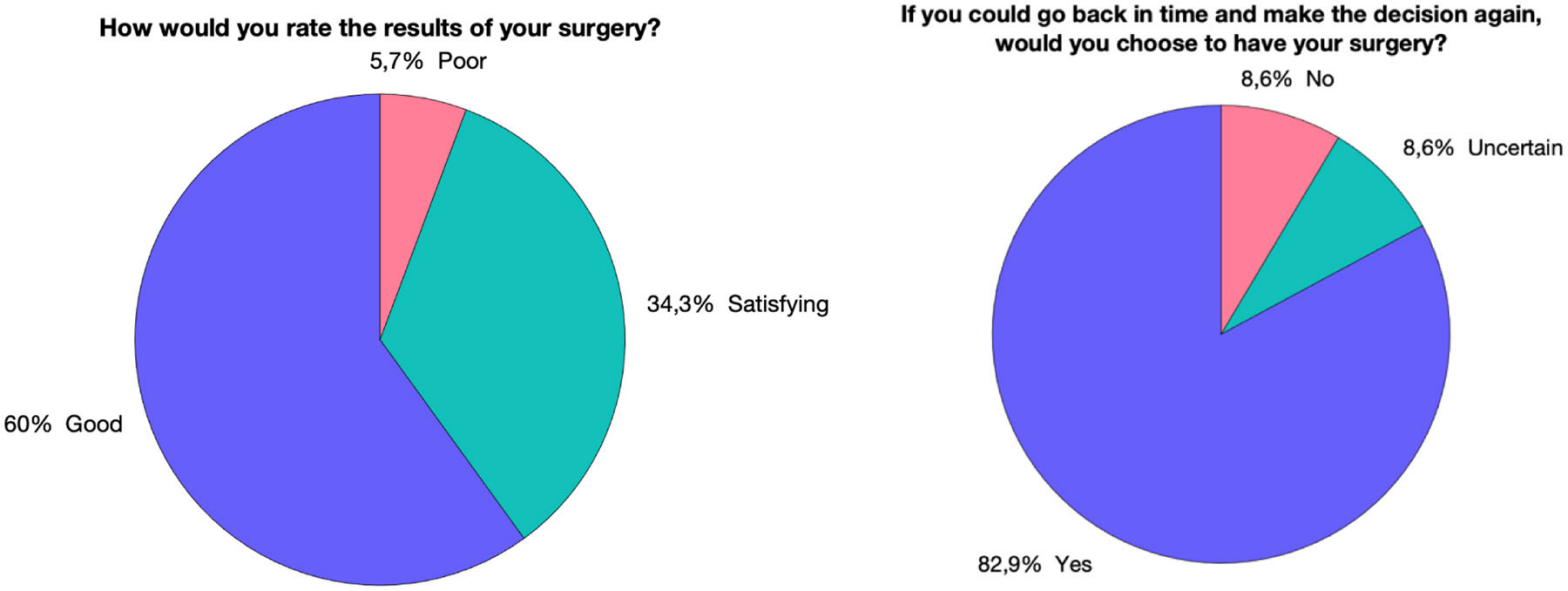
Assessment of the patient satisfaction survey.

The patient satisfaction survey revealed that 94.3% of patients were satisfied with the results of the operative treatment, with 82.9% indicating they would opt for the same treatment again (Figure 8).

### Range of motion and thigh and calf circumference

The thigh circumference 20 cm above the upper patella pole was significantly smaller in the operated leg compared to the contralateral leg (56.8 ± 4.9 cm vs. 57.6 ± 5.3 cm; *p* < 0.005), with an average difference of 0.75 cm. No significant differences were observed at 10 cm above the patella or in maximum calf circumference. The average range of motion for the operated knee was 4.7° (±4.1)/0°/125.8° (±6.4), which was significantly reduced in both extension (*p* = 0.03) and flexion (*p* = 0.03) compared to the contralateral knee, which measured 6.29° (±3.7)/0°/127.9° (±7.4).

### Gait analysis

The patient cohort demonstrated a significantly reduced self‐selected walking speed compared to healthy controls (0.98 ± 0.18 vs. 1.17 ± 0.17 m/s, *p* < 0.01).

Analysis of the VGRF parameters revealed significantly higher minimum values for both the operated and contralateral legs in the patient cohort compared to the legs of healthy controls (0.88 ± 0.10 and 0.87 ± 0.11 BW vs. 0.78 ± 0.07 BW, *p* < 0.01).

In the sagittal plane knee kinematics, the operated and contralateral legs in the patient cohort exhibited a significantly lower peak knee angle (PKA) during the stance phase compared to the healthy control group (9.58 ± 7.04° and 13.49 ± 6.88° vs. 17.66 ± 4.61°, *p* < 0.01). Additionally, the operated legs showed a significantly lower PKA compared to the contralateral legs (9.58 ± 7.04° vs. 13.49 ± 6.88°, *p* < 0.01).

In the analysis of knee kinetics during gait, the operated and contralateral legs of the patient cohort displayed significantly lower maximum knee extension moments compared to the healthy control group (0.11 ± 0.21 and 0.23 ± 0.19 Nm/kg vs. 0.42 ± 0.17 Nm/kg, *p* < 0.01). Furthermore, the operated legs had a significantly lower maximum knee extension moment compared to the contralateral legs (0.11 ± 0.21 vs. 0.23 ± 0.19 Nm/kg, *p* < 0.01).

**Figure 9:**
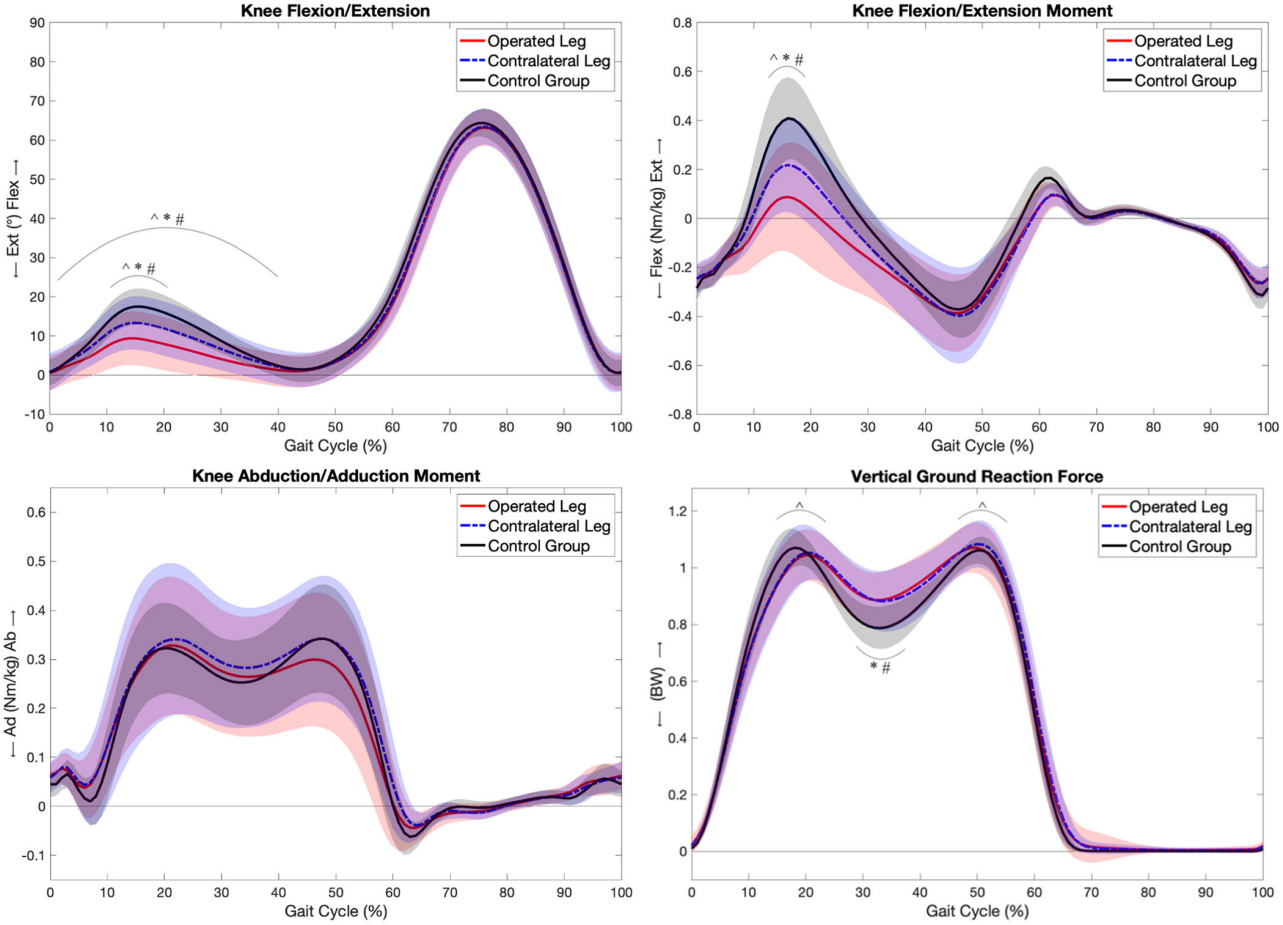
Knee kinematics, knee kinetics and vertical ground reaction force parameters during the gait cycle of the operated leg, the contralateral leg and the healthy control group. Coloured range: mean ± 1 standard deviation; ^: significant difference between operated leg & contralateral leg, *: significant difference between operated leg & control group, #: significant difference between contralateral leg & control group.

Detailed results for spatiotemporal parameters, knee kinematics, knee kinetics and VGRF parameters during the gait cycle are presented in Table 5 and Figure 9.

**Table 5:** Results of the spatiotemporal parameters, knee kinetics, knee kinematics and the vertical ground reaction force parameters of the operated leg, the contralateral leg and the control group.

	Operated leg	Contralateral leg	Control group	*p* Value
Spatiotemporal parameters				
Step length (m)	0.6 (±0.08)	0.59 (±0.08)	0.67 (±0.06)	**0.004** *
				<**0.001** **
				<**0.001** ***
Step width (m)	0.13 (±0.05)	0.13 (±0.05)	0.13 (±0.03)	0.750*
				0.971**
				0.963***
Stance time (s)	0.83 (±0.08)	0.83 (±0.09)	0.75 (±0.06)	0.609*
				<**0.001** **
				<**0.001** ***
Swing time (s)	0.4 (±0.03)	0.4 (±0.03)	0.39 (±0.03)	0.684*
				**0.044** **
				**0.030** ***
Self‐selected speed (m/s)	0.98 (0.18)		1.17 (±0.17)	<**0.001**
Knee kinematics				
Peak knee flexion angle (°) (Stance phase)	9.58 (±7.04)	13.49 (±6.88)	17.66 (±4.61)	<**0.001** * <**0.001** ** **0.004** ***
Range of motion (°) (Stance phase)	17.84 (±11.36)	25.21 (±11.72)	33.43 (±8.76)	<**0.001** * <**0.001** ** **0.001** ***
Knee kinetics				
Maximum extension moment (Nm/kg)	0.11 (±0.21)	0.23 (±0.19)	0.42 (±0.17)	<**0.001** * <**0.001** ** <**0.001** ***
1. Maximum abduction moment (Nm/kg)	0.34 (±0.14)	0.36 (±0.16)	0.33 (.09)	0.657* 0.841** 0.472***
2. Maximum abduction moment (Nm/kg)	0.31 (±0.13)	0.35 (±0.13)	0.35 (±0.11)	0.179* 0.222** 0.837***
Ground reaction force				
1. Maximum (BW)	1.06 (±0.09)	1.08 (±0.09)	1.08 (±0.07)	**0.022** * 0.327** 0.456***
2. Maximum (BW)	1.08 (±0.08)	1.09 (±0.08)	1.07 (±0.05)	**0.007** * 0.893** 0.530***
Minimum (BW)	0.88 (±0.1)	0.87 (±0.11)	0.78 (±0.07)	0.175* <**0.001** ** <**0.001** ***

*Note*: Bold values indicate statistical significance.

* Comparison between operated leg and contralateral leg.

** Comparison between operated leg and control group.

*** Comparison between contralateral leg and control group.

## DISCUSSION

The main findings of the present study demonstrate that MABCI results in significant improvements in PROMs for individuals with osteochondral defects of the knee at a mean follow‐up of 42.6 months. Specifically, the IKDC score improved from 56.6 preoperatively to 73.1 postoperatively, indicating a substantial enhancement in knee functionality from the patients' perspective. Additionally, all subscales of the KOOS score showed significant postoperative improvements, particularly in pain, symptoms and ADL.

Alongside these favourable subjective outcomes, the study identified postoperative gait abnormalities, indicating that biomechanical functionality may remain impaired. These objective gait disturbances highlight the complexity and multifaceted nature of functional recovery following MABCI. When compared to the existing literature, the findings are consistent with previous studies that report favourable outcomes following ABCI. Ochs et al. observed an increase in the IKDC score from 50.5–78.4 in their analysis of 26 patients with a mean follow‐up of 39.8 months [30]. Similar to the present study, they performed a combination of MACI for cartilage treatment and used autologous corticocancellous bone plugs for bony reconstruction. However, they utilised corticocancellous bone plugs from the iliac crest and used a scaffold‐based MACI. Andriolo et al. observed an even greater increase in the IKDC score, from 39.9 to 85.0 postoperatively [3]. They analysed 29 patients with a final follow‐up of 143.6 months but also reported four early failures. They performed a MACI with a hyaluronan‐based scaffold and used an autologous cancellous bone graft from the ipsilateral tibia for the bony reconstruction, which was performed in the first surgery of the two‐step procedure. Könst et al. demonstrated an increase in the IKDC score from 35.0 to 57.0 in their study analysing six patients at a 12‐month follow‐up using a combination of an autologous corticocancellous bone graft from the femur condyle and a gel‐based autologous chondrocyte implantation [23]. Maus et al. observed an increase in the IKDC score from 38.4 to 66.1 postoperatively analysing 13 patients at a 36‐month follow‐up [26]. The analysed patient cohort had a mean lesion size of 8.1 cm^2^, which is larger compared to the present study. Nevertheless, 91.7% of the patients rated their postoperative result as at least satisfying, which is similar to the results of the present study. Additionally, a recently published systematic review identified significant improvements in PROMs after ABCI, which aligns with the results observed in this study [31]. Another recent study comparing clinical outcomes of patients treated with MACI and MABCI using data from the German Cartilage Registry 24 months postoperatively showed nearly identical results of the analysed cohort of the patients treated with MABCI regarding the postoperative subscales of the KOOS Score in comparison to the results of the present study [43].

There are very few studies analysing postoperative gait biomechanics following autologous chondrocyte implantation. To our knowledge, no studies have yet examined gait biomechanics after combined treatment with autologous bone grafting [39]. Ebert et al. analysed the gait biomechanics of 61 patients treated with MACI for chondral lesions of the knee at 3, 6 and 12 months postoperatively and compared them to a healthy control group [10]. Similar to the patient cohort of the present study, the analysed patients exhibited a slower self‐selected walking speed compared to the healthy control group, but this difference was observed only at 3 months postoperatively and not at 6 or 12 months postoperatively. This may indicate that self‐selected walking speed is more affected in patients undergoing MABCI than by ACI alone. Similarly to the present study, the analysed patients showed no difference in the peak VGRF and a reduced knee extension moment during the stance phase. Also, similar to the present study, significant differences in knee flexion angle at heel strike (HS), midstance and change in knee flexion angle from HS to weight acceptance between control subjects and the patient groups at three and 6 months postsurgery could be observed, but with no difference at 12 months. The analysed patient cohort showed a significantly lower peak knee flexion angle (PKA) and a significantly reduced range of motion in knee flexion and extension during the stance phase of the operated leg and the contralateral leg compared to the healthy controls at 42.6 months of follow‐up. A possible explanation for the reduced PKA could be quadriceps weakness, which was not assessed but may play a contributing role. Müller et al. showed that patients showed significant strength deficits on the operated extremity 4 years after ACI [28]. Ebert et al. were also able to demonstrate that despite having recovered their knee flexor strength, patients still showed a reduced knee extensor strength profile at a 5‐year follow‐up after ACI [11]. Although quadriceps strength was not assessed, an indication for a present quadriceps weakness in the analysed patient cohort could be the significantly smaller thigh circumference 20 cm above the patella in the operated legs compared to the contralateral legs.

There are several studies that observed altered gait biomechanics after ligament reconstructions of the knee [22, 32]. A reduced PKA could be observed in several studies after anterior cruciate ligament reconstruction and multiple ligament reconstruction [15, 16, 34]. Patients following total knee arthroplasty also exhibit reduced knee joint flexion during the stance phase [25]. This is analogous to the present results and may suggest that similar adaptive mechanisms are involved. In addition to quadriceps weakness, perioperative damage of articular and periarticular structures like receptors, ligaments, muscles and tendons can result in sensory impairments that impact proprioception and modify neuromuscular activity. Moreover, the analysed patients experienced a significant reduction in pain levels postoperatively, though they were not completely pain‐free. The presence of pain or reflex inhibition may additionally affect movement patterns [38].

The gait analysis revealed a significantly higher minimum VGRF during the stance phase in the patient cohort compared to healthy controls, indicating reduced physiological unloading. This increased load with each step could contribute to osteochondral deterioration and potentially accelerate osteoarthritis progression. Notably, this abnormal loading pattern was observed in both operated and contralateral legs. Additional gait abnormalities, such as decreased PKA and restricted knee extension moment, were also present bilaterally. This is consistent with previous studies analysing gait patterns after knee joint surgery and implies that a bilateral adaptation of the gait pattern occurs postoperatively [6]. This suggests that this is a multifactorial process in which potential quadriceps weakness and various neurophysiological mechanisms play a role [4].

The clinical implications of these findings are considerable. PROMs are invaluable for capturing the patient's perspective on the success of interventions, offering insights that are not always apparent through clinical or biomechanical assessments. In the present study, PROMs provided a clear indication of the patients' perceived improvement. The observed gait abnormalities suggest that biomechanical assessments should also be routinely integrated into postsurgical evaluations. Altered gait biomechanics, as reflected by joint angles, joint moments and ground reaction forces, can notably change the stress distribution in the cartilage and trigger a degenerative process, thus serving as a mechanical contributing factor to the development of osteoarthritis [9].

While the present study offers data on PROMs and postoperative gait patterns of MABCI, it is important to acknowledge certain limitations. The relatively small sample size and retrospective design may affect the generalisability of the findings. Additionally, one patient was excluded due to revision surgery requiring conversion to a customised metal implant, which can be considered a clinical failure. The high rate of participants who were lost to follow‐up is another limitation of this study. Furthermore, the follow‐up period, although sufficient to assess mid‐term outcomes, may not capture the full scope of long‐term functional recovery. Another limitation of this study is the heterogeneity of the patient cohort. Three patients underwent a concomitant high‐tibial osteotomy, while five patients received the MABCI procedure in the patellofemoral region. Despite all patients undergoing the same standardised MABCI procedure at a single academic medical centre, this heterogeneity may limit the generalisability of the biomechanical findings. Future research should focus on larger, multicentre studies with extended follow‐up periods to comprehensively evaluate both PROMs and biomechanical outcomes over the long term. Additionally, further investigation into the integration of PROMs with advanced biomechanical assessments is necessary to provide more insight into postoperative recovery and refine treatment protocols. In conclusion, the present study confirms that MABCI is an effective treatment for osteochondral defects of the knee, as evidenced by significant improvements in PROMs. The observed postoperative gait abnormalities highlight the importance of a multifaceted approach to patient assessment, combining both subjective measures and objective biomechanical evaluations to fully evaluate the outcomes of MABCI surgery.

## AUTHOR CONTRIBUTIONS

**Stephan Oehme**: Conceptualisation; formal analysis; investigation; methodology; project administration; funding acquisition; supervision; writing—­original draft; review and editing. **Danko Dan Milinkovic**: Formal analysis; writing; review and editing. **Azzurra Paolucci**: Writing—original draft; review and editing. **Sophie Krafzick**: Patient recruitment; data collection; data extraction; formal analysis; statistical analysis; review and editing. **Stephen Fahy**: Formal analysis; statistical analysis; proofreading; writing; review and editing. **Philipp Damm**: Execution of data analysis algorithms; formal analysis; supervision; review and editing. **Tobias Winkler**: Supervision; interpreting the statistical results; review and editing. **Tobias Jung**: Clinical coordinator; patient recruitment; funding acquisition; supervision; review and editing. **Benjamin Bartek**: Clinical coordinator; patient recruitment; supervision; writing; review and editing.

## CONFLICT OF INTEREST STATEMENT

The authors declare no conflict of interest.

## ETHICS STATEMENT

The study was approved by the local ethics committee (Ethikkommission der Charité‐Universitätsmedizin Berlin, approval‐Nr: EA4/220/20).

## Data Availability

The data are available from the corresponding author upon reasonable request.
